# Investigation of Soft Magnetic Material Fe-6.5Si Fracture Obtained by Additive Manufacturing

**DOI:** 10.3390/ma15248915

**Published:** 2022-12-13

**Authors:** Anton V. Agapovichev, Alexander I. Khaimovich, Yaroslav A. Erisov, Mikhail V. Ryazanov

**Affiliations:** 1 Engine Production Technology Department, Samara National Research University, 34 Moskovskoye Shosse, 443086 Samara, Russia; 2 Metal Forming Department, Samara National Research University, 34 Moskovskoye Shosse, 334086 Samara, Russia; 3 Samara Federal Research Center of the Russian Academy of Sciences, 3A Studencheskiy Pereulok, 443001 Samara, Russia; 4 Novolipetsk Steel Company (NLMK), 40, Bolshaya Ordynka Str., Bldg 3, 119017 Moscow, Russia

**Keywords:** additive manufacturing, selective laser melting, magnetic material Fe-6.5Si, metal powder, mechanical properties, microstructure

## Abstract

The freeform capability additive manufacturing (AM) technique and the magnetic efficiency of Fe-6.5Si steel have the potential for the development of electromechanical component designs with thin body sections. Moreover, the directional anisotropy of the material, which is formed during growth, improves the magnetic and electrical properties of Fe-6.5 wt%Si. We obtained the range of optimal technological modes of Laser Power Bed Fusion process (volume energy density (VED) of 100–140 J/mm^3^, scanning speed of 750–500 mm/s) to produce the samples from Fe-6.5 wt%Si powder, but even at the best of them cracks may appear. The optical microscopy and SEM with EDX analysis of the laser-fabricated structures are applied for investigation of this phenomena. We detected a carbon content at the boundaries of the cracks. This suggests that one of the reasons for the crack formation is the presence of Fe_3_C in the area of the ordered $\alpha ’\mathrm{FeSi}\;\left(B2\right)+{\mathrm{Fe}}_{3}\mathrm{Si}\left(D0_{3}\right)$ phases. Quantitative analysis based on crack initiation criteria (CIC) showed that the safe level of internal stresses in terms of the CIC criteria in the area of discontinuities is exceeded by almost 190%. Local precipitates of carbides in the area of cracks are explained by the heterogeneity and high dynamics of temperature fields, as well as the transfer of substances due to Marangoni convection, which, as a result, contributes to a significant segregation of elements and the formation of precipitate phases.

## 1. Introduction

Nowadays the increasing application of electrical components has led to the building of electrical machines with improving performances: electric transformers, electric motors, electric generators, and inductive filters are more and more required. It is vitally important to have the possibility to create and produce machines able to convert energy in an economically convenience way, which depends on several aspects: the most significant is the use of high performing magnetic materials [1,2]. Concerning materials, silicon steels constitute one of the most important classes of soft magnetic materials used in magnetic applications [3,4,5]. For more detail, there is good reason to mention that the excellent electromagnetic properties [6] combined with the proper electrical resistance are guaranteed for FeSi steels with a Si content of within 2 wt.% and 7 wt.% [6,7], which are successfully accepted as reference materials for ferromagnetic cores of electric motors, generators, electrical transformers, etc. [8]. The actually adopted process designed to produce ferromagnetic is based on the superposition of FeSi thin foils coated by a dielectric material [1]: this will result in inhibiting the induced currents circulation path, thus cutting eddy current losses [9]. Technological limits are provided by such a process. As a matter of fact, FeSi steel with 6.5 wt.% Si offers the best soft magnetic proprieties [10] such as high magnetic saturation, low magneto-crystalline anisotropy, low magnetostriction and, above all, high electrical resistivity [11].

The previous important breakthrough in soft magnetic materials research occurred in the decade of 1970 [12]. It is connected with the development of nanocrystalline ribbons, a material displaying different degrees of crystallinity including amorphous state. Its main advantage is the ability to receive the optimal content of silicon, Fe-6.5 mass % Si, for the best soft magnetic properties (for instance, almost zero magnetostriction). It helps to avoid the problem of brittleness. The process consists of a rapid quenching of the molten material on a spinning wheel, resulting in formation of a ribbon [13] which can be manipulated for obtaining a desired microstructure and texture [14]. However, silicon steels with Si content higher than 4.5% are intrinsically brittle: due to their low machinability, it is not possible to cold reduce them down to thickness values (0.30–0.60 mm) required by the above-described process [15]. The brittleness of the high Si steels is mainly based on their ability to form phases with ordered structures during cooling. The annealing process, required for optimization of the magnetic properties, generally leads to final brittle materials. The ordered phases can be partly suppressed if the alloy is cooled fast enough from a high temperature kinetically trapping the disordered solid solution or amorphous state [16,17].

Later, some alternative processes have been studied aimed at creating parts in other ways that are not suitable for production by traditional way (e.g., [18]): rapid quenching route capability has been exploited to prevent the order–disordered phase transition [19]; chemical vapor deposition (CVD) [20] has been also exploited, aimed to deposit Si by thermal diffusion over low Si content coils. Other process routes include direct powder rolling, strip casting, physics vapor deposition (PVD) or spray forming [21]. All these methods have proven too expensive to be industrially sophisticated; therefore, they are nowadays considered of poor practical use. For many years, additive manufacturing (AM) has been imposing itself as a solid technology to produce metallic material components on industrial scale. Various parts of electrical devices were obtained with the help of AM [22,23,24]: mechanical and thermal management assemblies, coils/windings, permanent magnets, stator/rotor packs.

It is worth noting that the Fe-based alloys play a vital role [25] in the industry. Recently, the role of additive manufacturing has been recognized as a promising alternative route for the production of FeSi magnetic components with a high Si content [9]. The high cooling rates associated with the laser melting process allow avoiding the typical disordered-ordered phase transition in FeSi steels. This also will allow optimizing the ferromagnetic cores geometry. Although the ability of AM to produce even more complex and efficient components is a goal of creating an innovative class of materials for ferromagnetic cores that can meet the complex demands from the electric traction industry and, in general, from electric propulsion transport [22].

A major goal in printing soft magnetic Fe–Si steels using additive manufacturing is to take advantage of the potential for complex geometric designs and site-specific grain control for a thin wall geometry that produced a strongly columnar grain structure [26].

The columnar grains grow perpendicular to the grain boundary by a solidification process involving dendrites and may be the cause of solidification cracking.

At present, the Powder Bed Fusion (PBF) is the most common AM technology in industrial sphere. The PBF technology mainly uses the laser source as an energy source. The components are fabricated by laser melting metal powders [27], directly from Computer Aided Design (CAD) models [28].

The purpose of this article is to study and determine the modes of laser PBF of soft magnetic steel Fe-6.5 wt%Si that provide a structure without pores and cracks. An in-depth analysis of the causes of crack formation is carried out using SEM-EDX microelement analysis in the crack area and a quantitative assessment of the excess of the permissible level of internal stresses based on crack initiation criteria (CIC) calculations.

## 2. Materials and Methods

In this paper, a study to determine optimal technological scanning parameters for the Fe-6.5Si magnetic steel to be processed by laser PBF. The objects of study are the Fe-6.5Si powder produced by Höganäs and the samples obtained from this powder by the laser PBF technology.

The Fe-6.5Si magnetic steel metal powder was used as an initial material. The morphology of surface powder and chemical composition was investigated by scanning electron microscope Tescan Vega and Energy Dispersive X-rays Spectrometer INCAx-act, correspondingly.

Samples were fabricated from Fe-6.5Si magnetic steel powder on a SLM 280HL 3D printer (SLM Solutions Group AG, Lubeck, Germany). This 3D printer prints using laser PBF technology, the essence of which is the layer-by-layer manufacturing of a part by fusing layers together [29]. Laser PBF processing was under argon protection to ensure that the oxygen content was below 300 ppm. For the related scanning strategy, selected rotation direction between layers was 67°, and the selection of laser power, scanning speed, hatch distance, and layer thickness are shown in Table 1. The determination of the optimal scanning parameters was carried out on proportional flat samples with dimensions (L × W × H) 70 × 2 × 15 mm. The study was carried out in accordance with the model of a fractional factorial experiment 6^2^ × 3^2^//36 transformed from 6^3^//36 by replacing the 1st column.

The density of the samples was determined in accordance with GOST (State Standard) 20018-74 (ST SEV 1253-78, ISO 3369-75) “Sintered Hard Alloys. Density De-termination Method Using a Laboratory Balance, Caliper, Measuring Cup and Pycnometer”.

To determine the non-melting area and the diameter of the pores, thin sections of the cross section of the samples were prepared. Etching of the samples was carried out by electrolytic method at room temperature for 5–10 s in an electrolyte of the following composition: 10 g of citric acid + 10 g of ammonium chloride + 1 l of water.

Microanalysis was carried out on an optical microscope METAM LV-41 in a bright field with ratio from 50 to 200 magnification. The processing of the obtained images of the microstructure was carried out in a specialized software product SIAMS.

Statistical analysis of the data obtained as a result of the experiments was carried out in the commercial software product STATISTICA 13.

## 3. Results and Analysis

### 3.1. Powder Distribution

**Figure 1:**
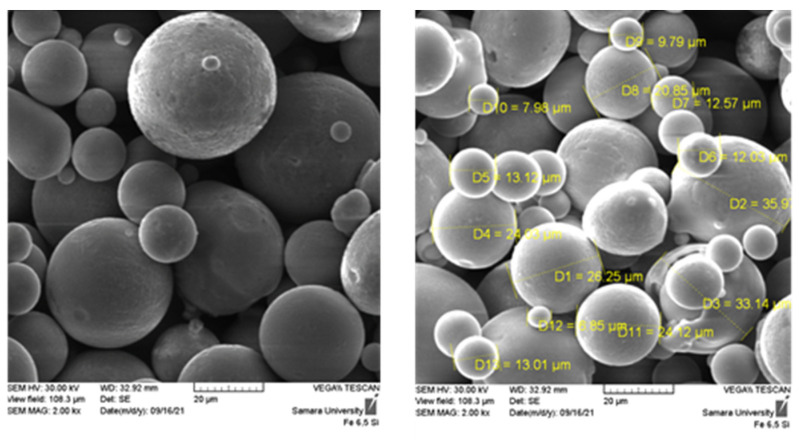
Particles of Fe-6.5Si magnetic steel powder.

The general view of the Fe-6.5Si powder produced by Höganäs particles is shown in Figure 1. Electron microscopic analysis showed that the powder particles mainly have a spherical shape (92%), which is typical for the method of obtaining powders by melt dispersion [30]. The particle size varies in the range of 5–40 microns. Plus and minus fractions are 0.5% and 1.5%, respectively.

Microspectral analysis showed that the chemical composition of the Fe-6.5Si magnetic steel powder complies with the manufacturer quality certificate (Si 6.3—6.7 wt.%, Fe—the rest).

### 3.2. Microstructural Characterization and Mechanical Property Tests

**Figure 2:**
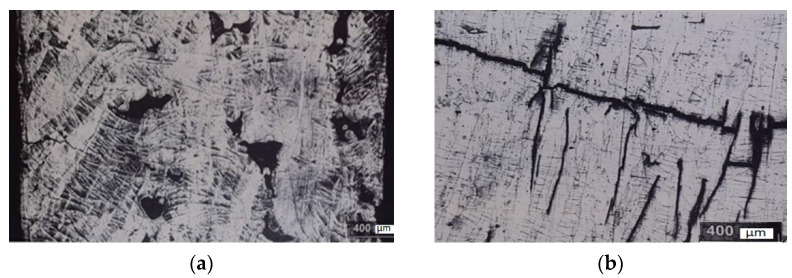
Fragments of the microstructure with typical defects of samples from Fe-6.7Si: (**a**) is a sample No. 4 (multiple lack of penetration over the entire cross section of the sample), (**b**) is a sample No. 34 (multiple cracks going in different directions).

Structure microanalysis revealed that the material of most samples, depending on the specific energy of fusion, has defects in the form of cracks extending from the surface to a depth of 1 mm and multiple non-melting areas. Figure 2 presents the typical defects of structure.

**Figure 3:**
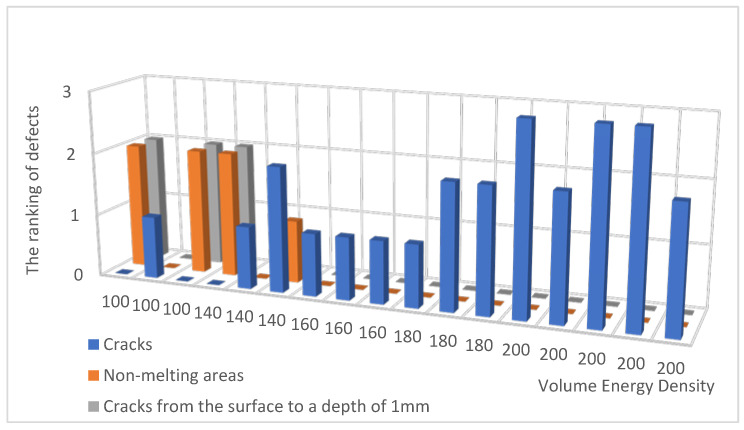
The histogram of defects from Table 2.

Samples from 17 experiments with the least number of defects were selected for further analysis. Data on the sample structure defects are given in Table 2. The table lines are arranged in order of increasing fusion energy density. The ranking of defects was carried out on a three-point scale for assessing the tendency to form cracks (one point is for a grid of small cracks, three points are for fully open cracks) and on a two-point scale to analyze the presence of non-melting areas (one point is for individual non-melting areas, two points are for significant non-melting areas). The symbol * in the table conventionally denotes undetected defects, which does not mean their actual absence. The histogram of defects is presented in Figure 3.

The influence of factors on the responses was assessed using the Pearson multiple correlation matrix, Table 3 (statistically significant correlation coefficients are in bold).

A pair correlation coefficient analysis shows that the greatest influence on the cracks formation and lack of penetration is exerted by the fusion power density R = 0.8 and R = −0.67, respectively. The laser power for the selected experiments is strongly related to the power density (R = 0.77), so its effect is similar to that of the density. The hatch distance and layer thickness for this sample of experiments (analysis Stage 1) does not have a significant value (−0.32 < R < 0.31). Deep cracks are strongly correlated with non-melting areas (R = 0.95), which most likely act as stress concentrators during solidification.

**Figure 4:**
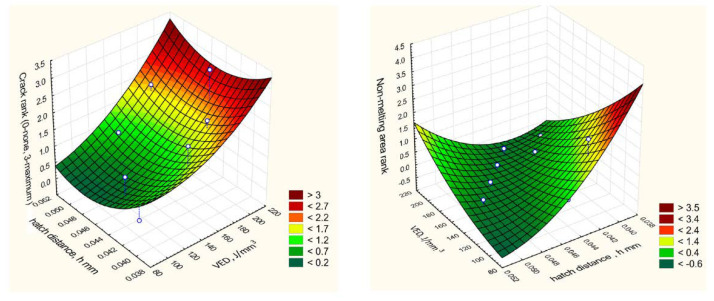
RSA Method for Determining the Area of Optimal Fe-6.5Si Alloying Modes.

**Figure 5:**
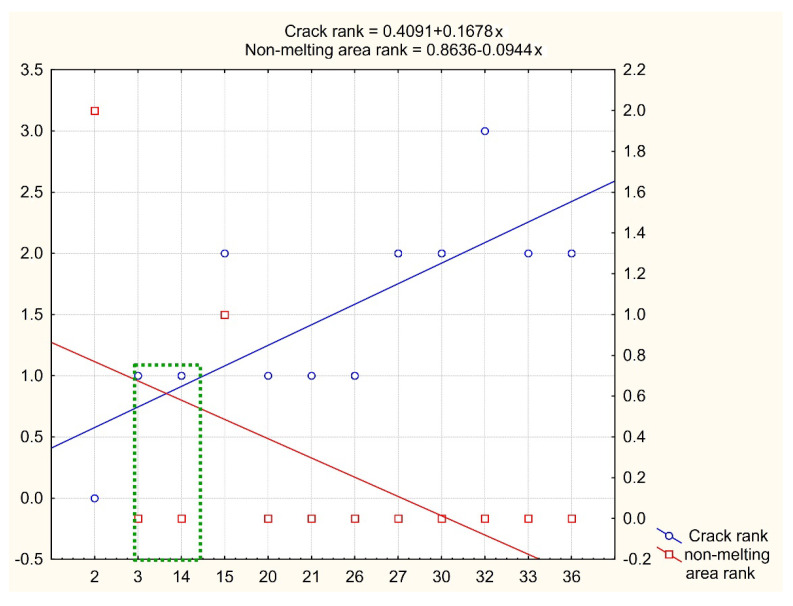
RSA Method for Determining the Area of Optimal Fe-6.5Si Fusion Modes.

The area of optimal modes was determined by the response surface analysis (RSA) method. The analysis results are shown in Figure 4 and Figure 5.

RSA shows that the best technological conditions lie in the range of volume energy density E = 100–140 J/mm^3^, with a hatch distance of about 0.05 mm and layer thickness of 0.05 mm.

Since the volume energy density has a dominant effect on the continuity of the sample structure, the area of preferred fusion modes was refined by the linear regression analysis method. The results of the analysis are presented in Table 4.

Regression analysis shows that with an increase in the volume energy density, the resistance to cracking decreases with a proportionality factor of 0.0222 (for a crack rank from 0 to 2). Sensitivity of the crack rank/non-melting area rank ratio = 1.52. This value was taken into account when assigning intervals for varying technological modes in the second series of experiments to determine their optimal values. To refine the definition domain for the second series of experiments, graphs of the trends of non-melting area and cracks in points were plotted depending on the experiments ordered by the specific fusion power density. The search area for optimal modes is highlighted with a dotted green box (Figure 5).

The area of optimal fusion modes highlighted in Figure 5 lies within power density of 100–140 J/m^3^; hatch distance of 0.044–0.05, scanning speed of 500–759 mm/s; layer thickness of 0.05 mm.

**Figure 6:**
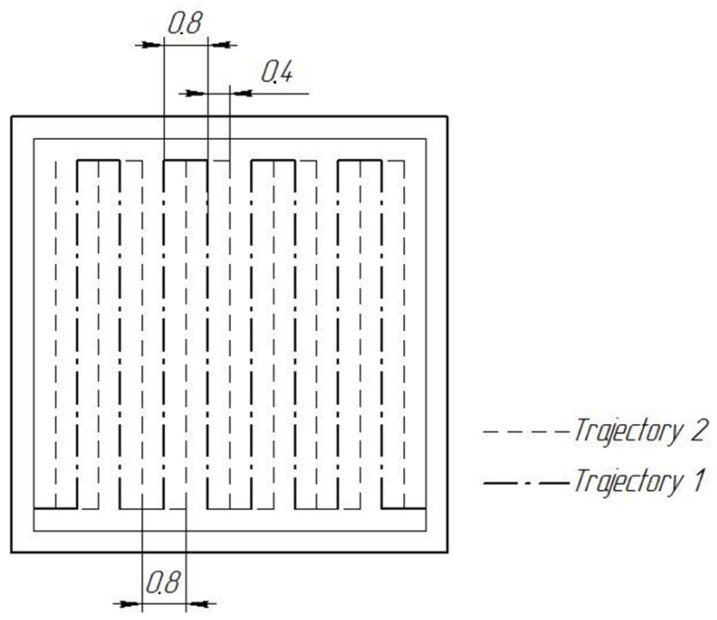
The sequence of track formation in the layer when printing samples.

**Figure 7:**
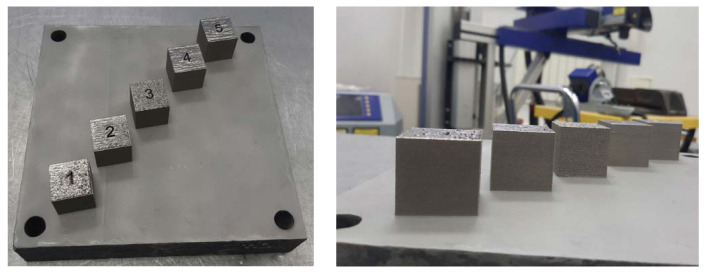
Grown samples on the build platform.

In order to obtain a defect-free structure at the second stage, in the recommended range of technological modes, small batches of proportional rectangular samples 10 × 10 × 10 mm were made by the SLS method, followed by control of the material structure and porosity. The 2^2^ × 3 experiment design obtained by D-optimal transformation from the 3^2^//9 [31] design is shown in Table 5. To study the effect on porosity of cooling time during fusion, two growth strategies were used, with and without track alternation (Figure 6). Samples grown on the build platform are shown in Figure 7.

The microstructural analysis of the samples for the presence of cracks, pores and non- melting areas established that cracks are observed on the sample grown in mode No. 3, individual microcracks are present along the edges of the samples. Residual porosity is typical for all samples. The maximum number of pores was observed in sample No. 3, the minimum in sample No. 2. The parameters of the technological mode No. 2 (Table 5) were taken as optimal. Considering Archimedes’ principle according to ASTM B311-17, density measurements are performed by measuring the 3D-printed cubic samples. For sample No. 2 manufactured under optimal conditions, the porosity was 0.99 ± 0.01. The measurement method error was determined by comparing the measured density with the theoretical one and was ±1%.

### 3.3. Microelemenl Analysis

**Figure 8:**
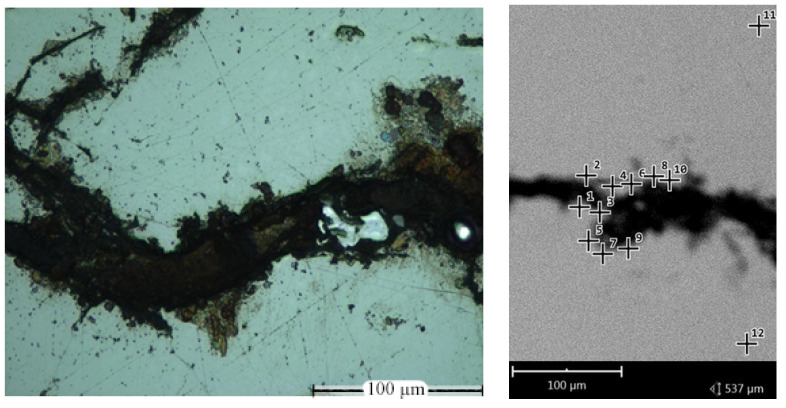
Elemental SEM-EDS analysis of the chemical composition along the edges of the crack: a is a crack fragment (sample no.), b is location of chemical composition analysis points.

As known, the Fe-Si6.5 system is prone to cracking. Traces of microcracks were observed in almost all alloyed samples. For a detailed clarification of the causes of this phenomenon, microelement SEM-EDS analysis was carried out. The study of the local chemical composition was carried out at points 1–10 located on the perimeter of the crack and at points 11, 12 located at a distance of more than 100 µm from the perimeter (Figure 8). The results of the study are presented in Table 6.

## 4. Discussion and Conclusions

**Figure 9:**
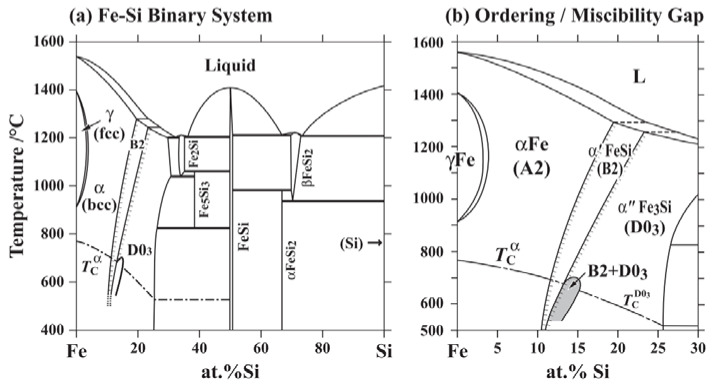
Phase diagrams of the Fe–Si binary system assessed by Kubaschewski from [33].

**Figure 10:**
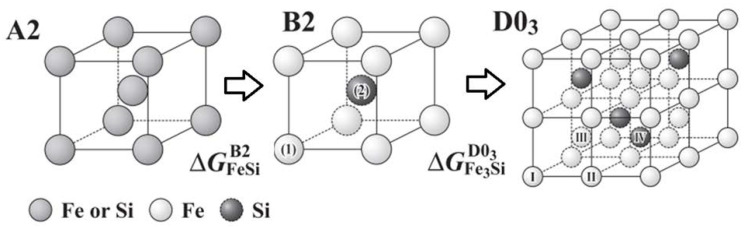
Atomic configurations of bcc phase, disordered A2, and ordered B2 and D03 (the image is reconstructed from [33]).

To explain the reasons for the crack formation, one should turn to the binary diagram of the phase state of Fe–Si. Figure 9 shows a phase diagram of the Fe–Si binary system assessed by Kubaschewski [32] which consists of liquid, γFe (A1), αFe (A2), α’FeSi (B2), α”Fe_3_Si (D03) and some intermetallic compound phases. Si stabilizes the αFe (A2) to form a γ-loop and causes two-step ordering from A2 to B2 and to D03 configurations as shown in Figure 10, with a Gibbs energy gain due to each ordering reaction. When silicon concentration increases to 12.5 at.% (6.45 wt.%), there will be two silicon atoms every eight cells on average. Thus, in a completely homogeneous solution, the phase will on average be Fe_7_Si. Upon increasing Si content, at 25 at.% (12.1 wt.%), there are four silicon atoms in eight cells and each silicon atom is surrounded by iron atoms up to the second nearest neighbor [12].

In an ordered structure of the B2 or B2 + DO_3_ types, upon deformation, the vacancies of atoms easily move to the grain boundary, since the elementary energy of such a movement in the periodic structure of the phase practically does not change. As a result, such dislocations accumulate at the grain boundary, which prevents their further movement; in this case, a significant internal stress arises, which is the cause of the structure brittleness.

B. Viala et al. found that movement of grain boundaries is impeded due to dislocation piling. A ductile-to-brittle transition is observed for T~1000 °C min^−1^, where long range B2 ordering takes place and the dislocation character changes from unitary to superlattice. The restricted glide and cross-slip capability of the dissociated superdislocations is consequently identified as the chief mechanism responsible for the buildup of internal stresses and eventual brittle fracture of the material [16,17]. The recovery process starts near 530 °C. As expected, the amount of silicon influences dislocation mobility through two main contributions, the increase in Peierls Nabarro force and the ordering, which raises stacking fault energy [16]. Decreasing overall ordering would increase dislocation mobility. However, unlike traditional technological processes for obtaining semi-finished products from Fe-6.5Si alloy, such as steer casting, chemical vapor deposition, the use of additive technologies is accompanied by heterogeneity and high dynamics of temperature fields, the transfer of substances from Marangoni convection, which contributes to significant phase segregation, is the reason for the precipitation of carbide-containing elements along the grain boundaries. This factor is the reason for the additional tendency of Fe-6.5 wt%Si to form cracks. This conclusion is confirmed by the high carbon content along the fracture perimeter (Figure 8, Table 6). This phenomenon should be considered in more detail. It is known that the presence of Si in the composition activates the action of C [34,35]. Faivre et al. investigated more extensively the composition domain where this carbide appears by studying the microstructure of samples rapidly cooled (1000 K/s) from the liquid state. Their results are shown in Figure 10, where a large composition domain for precipitation of the iron-silico-carbide from the liquid state is seen. The broken line reported in their figure is the eutectic line as assessed by Schiirmann and Hirsch, [36] and the full lines delineate the composition domain where both cementite and iron-silico-carbide were observed in fully solidified.

**Figure 11:**
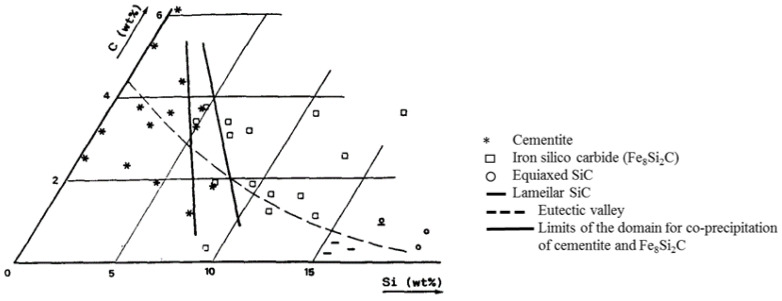
Variation with composition of the nature of the carbide phase observed from rapidly cooled samples from the liquid state (from [35]).

According to the Fe-Si-C ternary diagram (Figure 11) and microelement analysis data, $\alpha ’\mathrm{FeSi}\;\left(B2\right)+{\mathrm{Fe}}_{3}\mathrm{Si}\left(D0_{3}\right)$ phases may be present in the crack area (Table 6), and due to the high local carbon content of the ${\mathrm{Fe}}_{3}C\left(cementite\right),\;{\mathrm{Fe}}_{8}{\mathrm{Si}}_{2}C$ and ${\mathrm{Fe}}_{3}C/{\mathrm{Fe}}_{8}{\mathrm{Si}}_{2}C$ eutectic. The interfacial interface region can introduce additional stress into the $\alpha ’\mathrm{FeSi}\;\left(B2\right)+{\mathrm{Fe}}_{3}\mathrm{Si}\left(D0_{3}\right)$ matrix and contribute to the formation of cracks.

To quantify the influence of the interfacial interface on the stress state, one can refer to the continuity preservation condition (CPC) and the crack initiation criteria CIC [37,38]. The first parameter, depending on the microelement composition, characterizes the dimensionless ratio of thermodynamic quantities at a local point at which destruction does not occur. The second parameter determines the amount of excess CPC at the fracture boundary compared to its safe value outside the fracture. CPC and CIC can be calculated from dependencies (1) and (2).

(1) $$CPC_{v/w}=\frac{1}{24.304}\frac{C_{{}_{\Omega }}^{v}+C_{{}_{\Omega }}^{w}}{\frac{n^{w}}{\alpha_{m}^{w}{\left(T_{m}^{w}\right)}^{3/2}}+\frac{n^{v}}{\alpha_{m}^{v}{\left(T_{m}^{v}\right)}^{3/2}}}\left(\frac{1}{\sqrt{T_{0}}}-\frac{1}{\sqrt{T_{m}^{v}}}\right),$$

(2) $$CPC_{v}=\frac{1}{24.304}\frac{C_{{}_{\Omega }}^{v}}{n^{v}}\alpha_{m}^{v}\cdot T_{m}^{v}\left({\left(\frac{T_{m}^{v}}{T_{0}}\right)}^{1/2}-1\right),$$

(3) $$CIC=\frac{\left|CPC_{v/w}-CPC_{v}\right|}{CPC_{v}}.$$

In dependencies (1)–(3): $CPC_{v}$ is the continuity preservation condition for the phase of matrix; $CPC_{v/w}$ is the continuity preservation condition for the interface area between matrix and selection phases; CIC is the crack initiation criteria; the relative value of exceeding the permissible level of CPC in the area of the phase interface; $C_{{}_{\Omega }}^{v},C_{{}_{\Omega }}^{w}$ is the isochoric molar heat capacities of the matrix phase and the selection phase; $T_{0}$ is the temperature of normal conditions, $T_{m}^{v}$ is the melting temperature of the matrix phase; $\alpha_{m}^{v},\alpha_{m}^{w}$ are the coefficients of linear expansion of the matrix phase and the selection phase; $n^{v},n^{w}$ is the total number of atoms in the chemical compound of the matrix phase and the selection phase according to the rule of the Magnus–Lindemann Equation (3) [39] (for FeSi *n^v^* = 2, for Fe_3_Si *n^v^* = 4, for Fe_3_C *n^w^* = 4).

**Figure 12:**
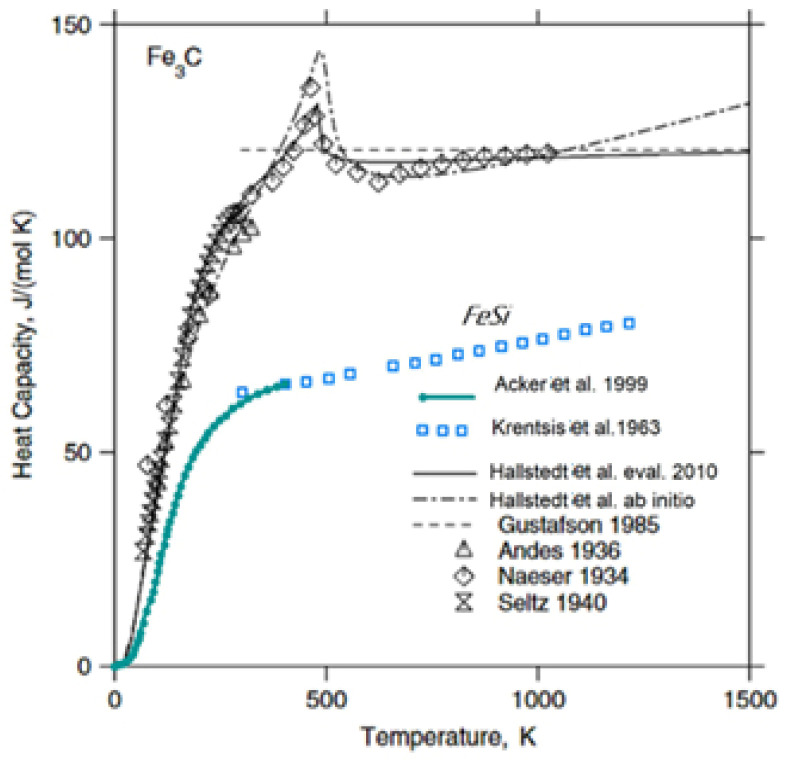
Heat capacity (C_p_) of Fe_3_C (reconstructed from [40]) and FeSi [41]. The solid line is calculated using the present thermodynamic description. The dash-dotted line shows the present theoretical ab initio-based calculation. The dashed line is from the thermodynamic description of Gustafson [42]. The symbols are from experimental measurements [43,44].

Calculations of the CPC and CIC parameters in the area of the Fe_3_C/FeSi and Fe_3_C/Fe_3_Si phase interface are presented in Table 7. The thermodynamic parameters of the phases from Table 7 were taken from the experimental data of other researchers. The frequently used Neumann–Kopp rule [39] cannot be used for FeSi and Fe_3_C, since the strong magnetic contribution to -Fe (bcc) leads to that which is far from zero and, as a consequence, a strongly temperature-dependent enthalpy (and entropy) of formation [40]. Figure 12 shows the curves of the isobaric heat capacity of FeSi and Fe_3_C as a function of temperature.

The calculated value of CIC = 1.92 for Fe_3_C/Fe_3_Si interface area (Table 7) shows that the permissible level of the CPC parameter, which characterizes the safe level of internal stresses in the area of discontinuity, is exceeded by almost 190%, which, in addition to the brittleness of the ordered $\alpha ’\mathrm{FeSi}\;\left(B2\right)+{\mathrm{Fe}}_{3}\mathrm{Si}\left(D0_{3}\right)$ phases, imposes an additional condition for the crack formation. It should be noted that the presence of B2 + DO_3_ phases is in the silicon concentration range of 6.45 wt.% (12.5 at.%; Figure 8).

## 5. Summary

AM makes it possible to obtain thin sections of parts of electrical products without pressure treatment. In addition, the directional anisotropy of the material, which is formed during growth, improves the magnetic and electrical properties of Fe-6.5wt%Si. High cooling rates that occur due to the high speed of the movement of the melt pool have reduced ordering, enhanced <100> out of the plane texture and increased coercivity. Nevertheless, as follows from experiments on the choice of optimal technological modes, at high values of the fusion energy, cracks appear in the grown samples. An in-depth analysis showed that the use of additive technologies is accompanied by heterogeneity and high dynamics of temperature fields, as well as the transfer of substances due to Marangoni convection, which, as a result, contributes to a significant segregation of elements and the formation of precipitate phases. The trace element analysis performed using SEM-EDS microscopy revealed a high carbon content at the boundaries of the detected cracks in the sample obtained using the L-PBF technology. This suggests that the cause of crack formation is the presence of Fe_3_C in the area of the ordered $\alpha ’\mathrm{FeSi}\;\left(B2\right)+{\mathrm{Fe}}_{3}\mathrm{Si}\left(D0_{3}\right)$ phases. The use of quantitative analysis based on the CIC criterion showed that in this case the safe level of internal stresses in terms of the CIC criterion in the area of discontinuities is exceeded by almost 190%.

## Figures and Tables

**Table 1:** The levels of experimental plan 6^2^ × 3^2^//36.

Change Range	Volume Energy Density VED, J/mm^3^	Laser Power P, W	Scanning Speed V, mm/s	Hatch Distance h, mm	Layer Thickness, t, mm
Min	100	80	500	0.04	0.04
Max	140	322	1000	0.05	0.05

**Table 2:** The experimental plan.

Sample №	Volume Energy Density, J/mm^3^	Hatch Distance, mm	Scanning Speed, mm/s	Layer Thickness, mm	Laser Power, W	Crack Points: 1 Is for Small Cracks, 2 Are for Cracks, 3 Are for through Cracks	Non-Melting Areas 1 Point Is for Individual, 2 Points Are for Multiple	Cracks from the Surface to a Depth of 1 mm
2	100	0.042	500	0.05	105	*	2	2
3	100	0.044	750	0.05	165	1	*	*
4	100	0.046	750	0.04	138	*	2	2
13	140	0.044	500	0.04	123	*	2	2
14	140	0.05	500	0.05	175	1	*	*
15	140	0.04	750	0.05	210	2	1	*
20	160	0.048	500	0.05	192	1	*	*
21	160	0.042	750	0.05	252	1	*	*
23	160	0.05	1000	0.04	320	1	*	*
26	180	0.046	500	0.05	207	1	*	*
27	180	0.05	750	0.05	338	2	*	*
30	180	0.042	1000	0.05	378	2	*	*
32	200	0.044	500	0.05	220	3	*	*
33	200	0.048	750	0.05	360	2	*	*
34	200	0.042	750	0.04	252	3	*	*
35	200	0.046	1000	0.04	368	3	*	*
36	200	0.04	1000	0.05	400	2	*	*
*—not tested								

**Table 3:** Pearson correlation matrix.

Factors/ Experiment Response Characteristics	h, mm	V, mm/s	t, mm	P, W	Crack Points	Non-Melting Areas	Cracks from the Surface to a Depth of 1 mm
VED, J/mm^3^	0.03	0.31	0.02	**0.77**	**0.80**	**−0.67**	**−0.63**
h, mm	1.00	−0.14	−0.13	0.05	−0.14	−0.24	−0.13
V, mm/s	−0.14	1.00	−0.27	**0.78**	0.39	−0.32	−0.34
t, mm	−0.13	−0.27	1,00	0.05	0.05	−0.32	−0.38
P, W	0.05	**0.78**	0.05	1.00	**0.66**	**−0.65**	**−0.62**
Crack points	−0.14	0.39	0.05	**0.66**	1.00	**−0.65**	**−0.70**
Non-melting areas	−0.24	−0.32	−0.32	**−0.65**	**−0.65**	1.00	**0.95**
Cracks from the surface to a depth of 1 mm	−0.13	−0.34	−0.38	**−0.62**	**−0.70**	**0.95**	1.00

**Table 4:** Regression analysis.

Regression Dependence	Correlation Coefficient-R, Error Probability at Determining Regression Coefficients-*p*	Sensitivity of Crack Rank/Non-Melting Area Rank in Relation to Power Density
Cracks rank = −2.111 + 0.0222⋅VED	R = 0.8 *p* < 0.009	1.52
Non-melting area rank = 2.758 − 0.0146⋅VED	R = 0.8 *p* < 0.004	

**Table 5:** The experiment plan 2^2^ × 3.

Sample №	Volume Energy Density VED, J/mm^3^	Scanning Speed V, mm/s
1	100	750
2	100	750
3	100	500
4	120	750
5	120	750
6	120	500
7	140	750
8	140	750
9	140	500

**Table 6:** Chemical composition of sample no. in the crack area, at.%.

Element/ Point Number	At Points along the Perimeter of the Crack										Outside the Perimeter	
	1	2	3	4	5	6	7	8	9	10	11	12
Fe	67.22	71.18	46.35	43.84	50.35	49.43	74.75	54.93	70.65	59.39	85.27	85.2
Fe_3_C + C	22.1	21.16	46.2	49.23	42.09	43.44	15.98	37.96	20.15	33.33	0	0
Si	10.69	7.67	7.44	6.92	7.56	7.13	9.27	7.11	9.2	7.29	14.73	14.44

**Table 7:** Calculations of the CPC and CIC parameters in the area of the Fe_3_C/FeSi and Fe_3_C/Fe_3_Si phase interface.

	FeSi	Fe_3_Si	Fe_3_C
Isobaric heat capacity $C_{\Omega }^{v},C_{\Omega }^{w}$, J/mol/K	60 [41]	24.58 [45]	110 [40]
Melting temperature T_m_, K	1300 ([33], Figure 1)	1600 [45]	1147 [Fe-Fe_3_C diagram]
CTE, K^−1^	1.62 × 10^−5^ [41]	1.3 × 10^−5^ [45]	1.90 × 10^−5^ [46]
CPC continuity condition	CPC for FeSi 0.0282 (Exp. 2)	CPC for Fe_3_Si 0.0069 (Exp. 2)	
CPC for interface	Fe_3_C/FeSi 0.0246 (Exp. 1)	Fe_3_C/Fe_3_Si 0.0201 (Exp. 1)	
CIC	0.127 (Exp 3)	1.92 (Exp 3)	
